# Characterization of prostatic cancer lesion and gleason grade using a continuous-time random-walk diffusion model at high b-values

**DOI:** 10.3389/fonc.2024.1389250

**Published:** 2024-05-24

**Authors:** Yurui Sheng, Huan Chang, Ke Xue, Jinming Chen, Tianyu Jiao, Dongqing Cui, Hao Wang, Guanghui Zhang, Yuxin Yang, Qingshi Zeng

**Affiliations:** ^1^ Department of Radiology, The First Affiliated Hospital of Shandong First Medical University & Shandong Provincial Qianfoshan Hospital, Jinan, Shandong, China; ^2^ Department of Radiology, Shandong Provincial Qianfoshan Hospital, Shandong University, Jinan, Shandong, China; ^3^ Magnenic Resonance (MR) Collaboration, United Imaging Research Institute of Intelligent Imaging, Beijing, China; ^4^ Department of Radiology, Shandong Public Health Clinical Center, Jinan, Shandong, China; ^5^ Department of Neurology, The Second Hospital of Shandong University, Jinan, Shandong, China

**Keywords:** the continuous-time random-walk diffusion (CTRW), prostatic cancer (PCa), chronic prostatitis (CP), grading group (GG), ADC

## Abstract

**Background:**

Distinguishing between prostatic cancer (PCa) and chronic prostatitis (CP) is sometimes challenging, and Gleason grading is strongly associated with prognosis in PCa. The continuous-time random-walk diffusion (CTRW) model has shown potential in distinguishing between PCa and CP as well as predicting Gleason grading.

**Purpose:**

This study aimed to quantify the CTRW parameters (α, β & Dm) and apparent diffusion coefficient (ADC) of PCa and CP tissues; and then assess the diagnostic value of CTRW and ADC parameters in differentiating CP from PCa and low-grade PCa from high-grade PCa lesions.

**Study type:**

Retrospective (retrospective analysis using prospective designed data).

**Population:**

Thirty-one PCa patients undergoing prostatectomy (mean age 74 years, range 64–91 years), and thirty CP patients undergoing prostate needle biopsies (mean age 68 years, range 46–79 years).

**Field strength/Sequence:**

MRI scans on a 3.0T scanner (uMR790, United Imaging Healthcare, Shanghai, China). DWI were acquired with 12 b-values (0, 50, 100, 150, 200, 500, 800, 1200, 1500, 2000, 2500, 3000 s/mm^2^).

**Assessment:**

CTRW parameters and ADC were quantified in PCa and CP lesions.

**Statistical tests:**

The Mann-Whitney U test was used to evaluate the differences in CTRW parameters and ADC between PCa and CP, high-grade PCa, and low-grade PCa. Spearman’s correlation of the pathologic grading group (GG) with CTRW parameters and ADC was evaluated. The usefulness of CTRW parameters, ADC, and their combinations (Dm, α and β; Dm, α, β, and ADC) to differentiate PCa from CP and high-grade PCa from low-grade PCa was determined by logistic regression and receiver operating characteristic curve (ROC) analysis. Delong test was used to compare the differences among AUCs.

**Results:**

Significant differences were found for the CTRW parameters (α, Dm) between CP and PCa (all P<0.001), high-grade PCa, and low-grade PCa (α:P=0.024, Dm:P=0.021). GG is correlated with certain CTRW parameters and ADC(α:P<0.001,r=-0.795; Dm:P<0.001,r=-0.762;ADC:P<0.001,r=-0.790). Moreover, CTRW parameters (α, β, Dm) combined with ADC showed the best diagnostic efficacy for distinguishing between PCa and CP as well as predicting Gleason grading. The differences among AUCs of ADC, CTRW parameters and their combinations were not statistically significant (P=0.051–0.526).

**Conclusion:**

CTRW parameters α and Dm, as well as their combination were beneficial to distinguish between CA and PCa, low-grade PCa and high-grade PCa lesions, and CTRW parameters and ADC had comparable diagnostic performance.

## Introduction

Prostatic cancer (PCa) ranks among the most prevalent urinary tract malignancies affecting male health, significantly impacting global male mortality rates and demonstrating an escalating annual incidence (1, 2). Accurate PCa diagnosis, malignancy assessment, and identification of clinically pertinent lesions are pivotal for optimizing therapeutic strategies and improving long-term survival (3). Presently, fine-needle aspiration biopsy, in conjunction with the Gleason scoring system serves as the established diagnostic gold standard for PCa. However, this method carries inherent risks of complications such as pain, bleeding, inflammation, and urinary difficulties, potentially expediting disease progression (4). Thus, finding an non-invasive and effective complementary means for the precise diagnosis and malignancy assessment of PCa is essential and holds significant value for clinical practice.

The apparent diffusion coefficient (ADC), derived from diffusion weighted imaging (DWI), serves to characterize the movement of the water molecular within the micro-environment (5, 6). Although ADC has been routinely used in the screening and diagnosis of PCa (7–9), it is based on the conventional DWI model, which assumes the Gaussian behavior of water diffusion within a homogeneous medium. However, heterogeneous structures in cancerous tissues can result in the non-Gaussian diffusion of water molecules (10). Given the considerable heterogeneity observed in PCa across pathological, clinical, and molecular domains (11, 12), the capacity of ADC to characterize PCa may be limited.

Recently, the continuous-time random walk (CTRW) diffusion model was developed and used to describing the heterogeneity of complex tissue microenvironments and tissue structures by providing three parameters, α, β, and Dm, which reflect temporal and spatial diffusion heterogeneity, and anomalous diffusion coefficient, respectively (12–14). Due to the advantages of the CTRW model in characterizing non-Gaussian diffusion behavior, it has been applied in recent studies for the assessment of glioma grading (15), the differentiation of high- and low-grade pediatric brain tumors (16), and the differentiation and prognostic assessment of benign and malignant breast lesions (17). Notably, no study has yet applied the CTRW model to prostate diseases.

This study aims to explore whether the CTRW diffusion model can reflect the microstructure heterogeneity of PCa, distinguish between CP and PCa, characterize PCa grade, and compare its efficacy with that of conventional ADC for diagnostic utility.

## Methods

### Patients

**Figure 1:**
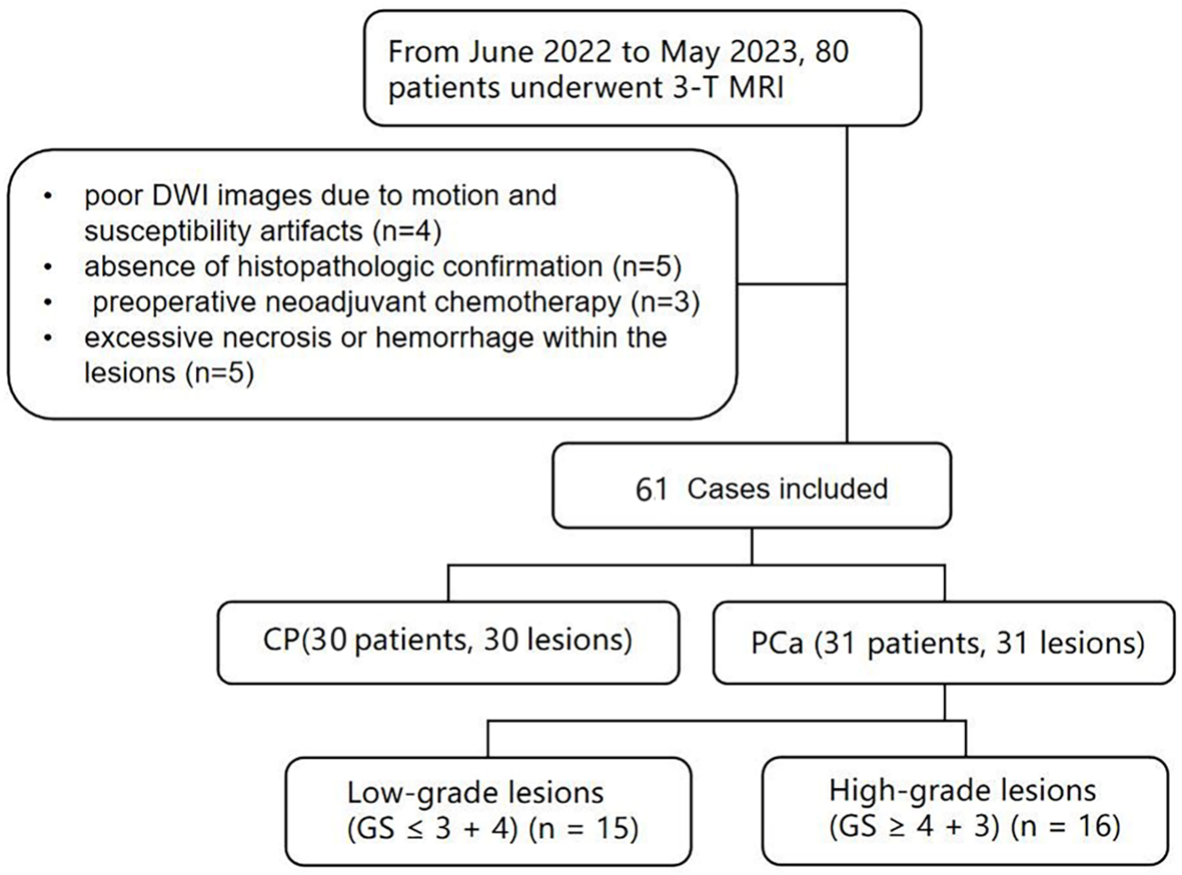
Flowchart shows patient selection process, CP, chronic prostatitis; PCa, prostate cancer; GS, Gleason score.

This study was approved by the Ethics Committee of the local institution, and written informed consent was obtained from all patients. From June 2022 to May 2023, 80 patients with suspected prostatic lesions based on clinical symptoms and ultrasonography, and without contraindications to MRI, were enrolled. Inclusion criteria were as follows: histopathological confirmed lesions; no therapy prior to MRI scan; MRI scans were performed within 2 weeks prior to surgery. Exclusion criteria included the following: poor DWI images due to motion and susceptibility artifacts (n=4); absence of histopathologic confirmation (n=5); preoperative neoadjuvant chemotherapy (n=3) and excessive necrosis or hemorrhage within the lesions (n=5). **Figure 1** shows the Flowchart of patient selection process. Thirty-one PCa patients undergoing prostatectomy (mean age 74 years, range 64–91 years), and thirty CP patients undergoing prostate needle biopsies (mean age 68 years, range 46–79 years).

### MRI data acquisition

All patients underwent MRI scans on a 3.0T scanner (uMR790, United Imaging Healthcare), including 1) axial fat suppression fast spin echo (FSE) T_1_-weighted imaging (T_1_WI); 2) axial fat suppression FSE T_2_-weighted imaging (T_2_WI); 3) DWI were acquired with 12 b-values 0_1_, 50_1_, 100_1_, 150_1_,200_1_, 500_2_, 800_3_, 1200_4_, 1500_4_, 2000_5_, 2500_8_, 3000_10_ s/mm^2^), where the subscripts represent the number of averages, TR/TE = 2425/57.3 ms, slice thickness = 3.5 mm, matrix size =112×112, scan time = 7min18s, field of view(FOV) =235×235mm^2^.

### Image analysis

All DWI data was transferred to the MATLAB (MathWorks, Inc., Natick, MA) for post processing.

The CTRW model was fitted by the following equation (14–16):


$$S/S_{0}=E_{\alpha }[(-{\left(bD_{m}\right)}^{\beta }]$$

where Dm is an anomalous diffusion coefficient, α and β are parameters related to temporal and spatial diffusion heterogeneity, respectively, and Eα is a Mittag-Leffler function.

The ADC was calculated for comparison using the following equation:


$$S/S_{0}=e^{b*ADC}$$

where S is the signal intensity acquired at b-values= 800 sec/mm^2^, S_0_ is the signal intensity in the voxel with b-values=0 sec/mm^2^.

### ROI analysis

Before determining the lesion locations for each patient, two independent radiologists (Z.Q.S and S.Y.R with 20 years and 5 years of experience in the field of prostate MRI, respectively) were only told that the patient had a prostate lesion, but had no pathological information. Only lesions with corresponding locations in both MRI and pathology were included for analysis. In cases where multiple lesions were observed in the prostate, the largest lesion with the highest grade was chosen as the target lesion for analysis. Thus only one target lesion was analyzed per patient.

Regions of interest (ROIs) were manually delineated on the slice with the largest area of solid tumor on the b = 1200 sec/mm^2^ DWI images using ITK-Snap software. Cystic components, necrotic areas, and hemorrhage areas were avoided. The ROIs were then copied to the corresponding Dm, α, β, and ADC maps for further analysis. Lesion size and lesion location were recorded.

### Histopathology analysis

Both prostatectomy and prostate needle biopsies were performed by urologists with 15 years of relevant experience. Ultrasound guided prostate needle biopsies through the rectum was performed by conventional 6-area 12-needle puncture. If there are suspicious lesions, 1 needle can be reused. Specimens were then collected into numbered vials and pathology scores were assigned to prostatectomy and prostate needle biopsies specimens by urogenital pathologists with 20 years of experience. PCa samples were graded according to the Gleason grading system (18), and the PCa group could be divided into two groups: low-grade cancer group (GS ≤ 3 + 4) and high-grade cancer group (GS≥4 + 3).

### Statistical analysis

All parameters were reported as mean ± standard deviations (SD). Normality test was performed using the Shapiro-Wilk test. Intra-class correlation coefficients (ICC) and their 95% confidence intervals (CIs) were calculated (ICC>0.80, excellent agreement; 0.61–0.80, good agreement; 0.41–0.60, moderate agreement; 0.21–0.40, poor agreement;<0.20, disagreement). The Mann-Whitney U test was used to evaluate the differences in CTRW parameters and ADC between PCa and CP as well as high-grade PCa (GG≥3) and low-grade PCa (GG ≤ 2). Spearman’s correlation assessed the association of GG with CTRW parameters and ADC. Binary logistic regression and receiver operating characteristic (ROC) curve analysis were employed to evaluate the diagnostic accuracy of ADC, individual CTRW parameters and their combinations (Dm, α and β; Dm, α, β, and ADC) for distinguishing PCa from CP and high-grade PCa from low-grade PCa. Furthermore, the area under the ROC curve (AUC), accuracy, sensitivity, and specificity were calculated. Delong test was used to compare the differences among AUCs. SPSS and MedCalc software was used for all statistical analyses with P-values< 0.05 considered as statistically significant.

## Results

### Patients and lesions

Demographics and clinical data of the 61 included patients (including CP and PCa) are shown in **Table 1**. Pathological characteristics of PCa patients, including GG and GS (Gleason score), are also shown in **Table 1**.

**Table 1:** Demographics and clinical data of the 61 included patients (including CA and PCa).

Variable	Data
Age: mean (range) CP (n=30)	74 (64–91) y
PCa (n=31)	68 (46–79) y
Serum PSA: mean ± SD (range) CP (n=30)	25.9 ± 18.7 (0.4–301.1) ng/ml
PCa (n=31)	9.8 ± 11.5 (0.2–35.7) ng/ml
Pathological grade of prostate cancer lesions	
GG 1/GS ≤6: number of tumors (%) tumors (%)	8 (25.8%)
GG 2/GS 3 + 4: number of tumors (%)	7 (22.6%)
GG 3/GS 4 + 3: number of tumors (%)	6 (19.4%)
GG 4/GS 8: number of tumors (%)	4 (12.9%)
GG 5/GS 9/10: number of tumors (%)	6 (19.4%)
MRI size of lesions: mean ± SD (range) mean? SD (range)	1.1 ± 0.5 (0.6–2.3) cm

CP, Chronic Prostatitis; PCa, Prostate Cancer; GG, grade group; GS, Gleason score; PSA, prostate-specific antigen; SD, standard deviation.

### Interobserver agreement of ADC, Dm, α, and β measurements

The ICCs with 95% confidence intervals for ADC, Dm, α, and β are shown in **Table 2**. The ICCs of all parameters were greater than 0.94, representing excellent interobserver agreement.

**Table 2:** ICC for the Parameters Measured by Two Radiologists.

Parameters	ICC	95% CI	P-value
α	0.947	0.895 ~ 0.974	<0.001***
β	0.976	0.952 ~ 0.989	<0.001***
Dm	0.985	0.970 ~ 0.993	<0.001***
ADC	0.998	0.998 ~ 0.999	<0.001***

ICC, intraclass correlation coefficient; ADC, apparent diffusion coefficient; CI, confidence interval; ***P-value<0.001.

### Comparison of CP vs. PCa and low-graded PCa vs. high-grade PCa

**Figure 2:**
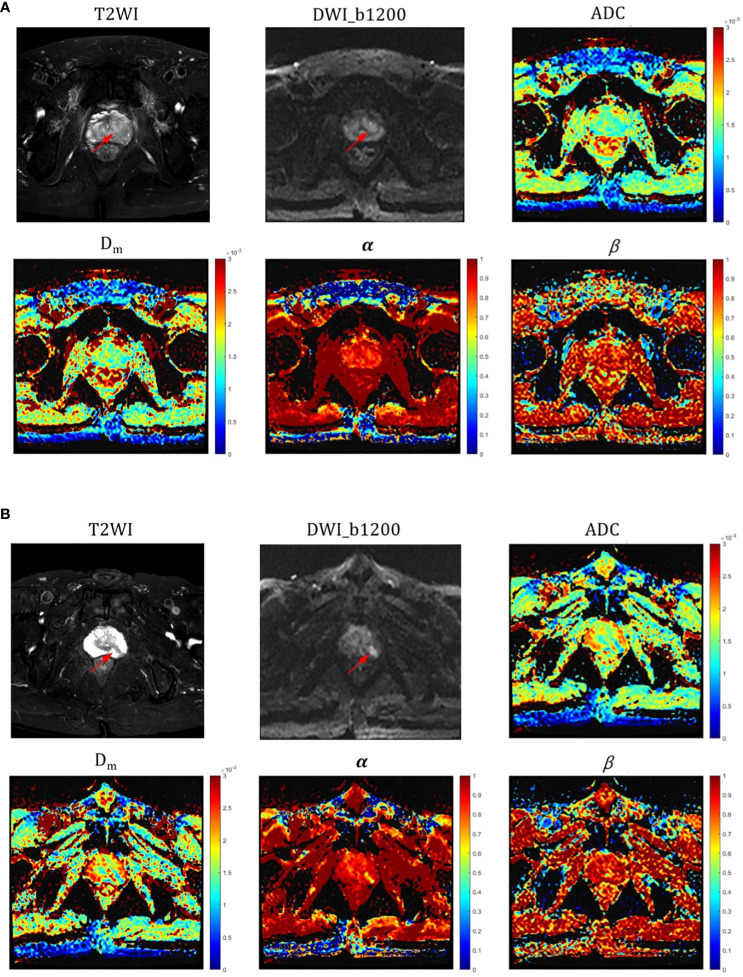
**(A)** Representative images (T_2_WI, DWI with b-value=1200, ADC, Dm, α and β) for a male patient with CP (red arrow pointing to); **(B)** Representative images (T_2_WI, DWI with b-value=1200, ADC, Dm, α and β) for a male patient with PCa (red arrow pointing to). ADC, α, and Dm values were all significantly different in CP and pCa, especially in α.

The α, Dm, and ADC exhibited significantly lower values in PCa lesions (0.603 ± 0.127, 0.819 ± 0.267 × 10–^3^ mm^2^/sec, and 0.852 ± 0.213 × 10–^3^mm^2^/sec, respectively) compared to those in CP lesions (0.743 ± 0.778, 1.352 ± 0.802 × 10–^3^ mm^2^/sec, and 1.274 ± 0.258 × 10–^3^mm^2^/sec, respectively) with all P-value< 0.001 (**Table 3**). However, no significant difference was observed in β values between the two groups (P=0.512). Representative images (T_2_WI, DWI with b-value =1200 sec/mm^2^, ADC, Dm, α, and β) for two male patients with CP and pCa are shown in **Figure 2**, respectively.

**Table 3:** Comparison of Dm, α, β, and ADC Among CA/PCa and Low-Graded/High-Grade PCa.

Lesions	α	β	Dm (×10–^3^ mm^2^/sec)	ADC (×10–^3^ mm^2^/sec)
CP(n=30)	0.743 ± 0.778	0.873 ± 0.519	1.352 ± 0. 802	1.274 ± 0.258
PCa(n=31)	0.603 ± 0.127	0.861 ± 0.089	0. 819 ± 0.267	0. 852 ± 0.213
P-value	<0.001***	0.512	<0.001***	0.011*
low-graded PCa(n=15)	0.691 ± 0.089	0.868 ± 0.109	1.133 ± 0. 704	1.043 ± 0.523
high-grade PCa(n=16)	0.586 ± 0.128	0.883 ± 0.049	0. 878 ± 0. 347	0.728 ± 0.212
P-value	0.024*	0.488	0.021*	0.039*

CP, Chronic Prostatitis; PCa, Prostate Cancer; ADC, apparent diffusion coefficient; *P-value<0.05, ***P-value<0.001.

**Figure 3:**
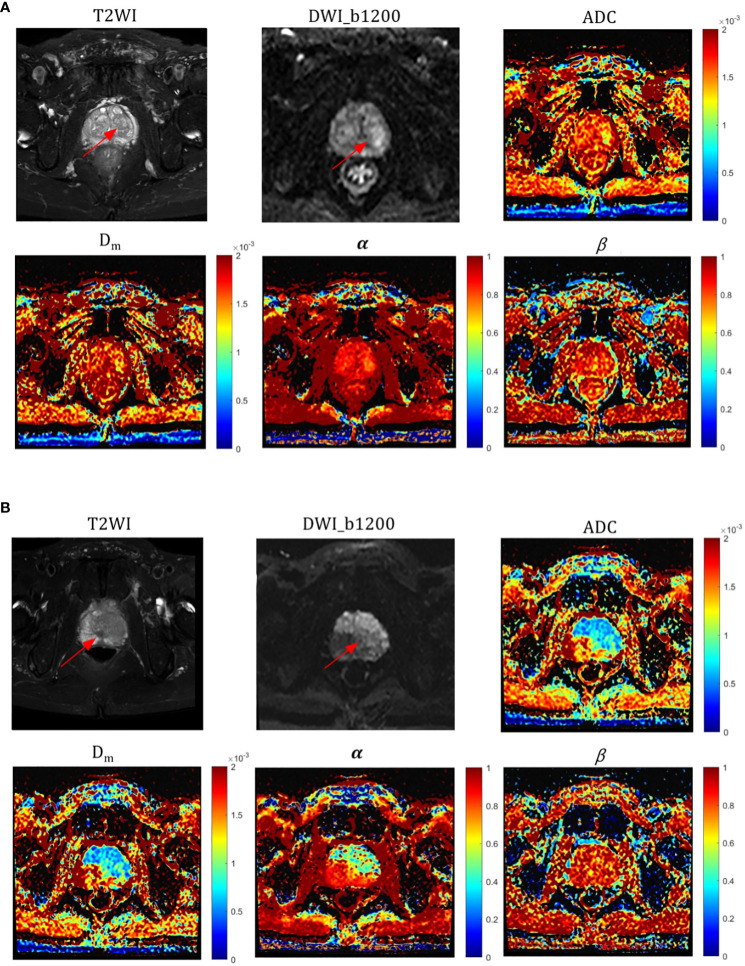
Representative images of T_2_WI, DWI, ADC, and CTRW parameters (red arrows represent lesions); **(A)** a patient with a low-grade (GG=2) pCa lesion and **(B)** a patient with a high-grade (GG=5) pCa lesion. ADC, α, and Dm values were significantly different in both high and low grade pCa.

The α, Dm, and ADC value were found to be significantly lower (P = 0.024, P =0.021, and P=0.039, respectively) in high-grade PCa lesions (0.586 ± 0.128, 0.878 ± 0.347 × 10–^3^ mm^2^/sec, and 0.728 ± 0.212 mm^2^/sec, respectively) compared to those in low-grade PCa group (0.691 ± 0.089, 1.133 ± 0.704 × 10–^3^ mm^2^/sec, and 1.043 ± 0.523 mm^2^/sec, respectively) (**Table 3**). Nevertheless, β values showed no significant difference between the two groups (P=0.488). Representative images (T_2_WI, DWI with b-value =1200, ADC, Dm, α, and β) for two male patients with low-grade and high-grade PCa are shown in **Figure 3**, respectively.

### Diagnostic performance for differentiation between CA and PCa

**Figure 4:**
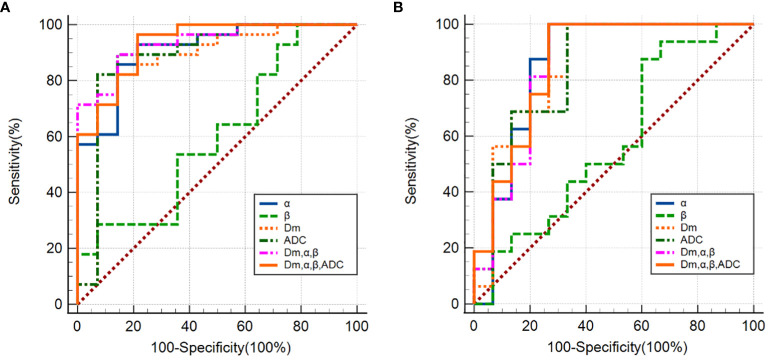
**(A)** ROC curves using single CTRW parameters (α, β, Dm), ADC and their combinations (Dm, α and β; Dm, α, β and ADC) differentiating between PCa and CP; **(B)** ROC curves using single CTRW parameters (α, β, Dm), ADC and their combinations (Dm, α and β; Dm, α, β and ADC) differentiating between low-grade and high-grade PCa.

The ROC analysis results are shown **Figure 4A** and **Table 4**. Among individual parameters, Dm yielded the highest sensitivity, specificity, and AUC (83.3%, 85.7%, and 0.89), while α exhibited the highest accuracy and sensitivity (88.1%, 83.3%) but relatively lower specificity and AUC (77.4%, 0.84). ADC provided the modest sensitivity, specificity, accuracy, and AUC (80.0%, 87.1%, 85.7% and 0.86, respectively). Combining parameters could obtain equivalent or higher AUC values, sensitivity, specificity and accuracy compared to individual parameters (Dm, α, β & ADC: 0.93, 90.0%, 87.1%, 90.5%; Dm, α, β: 0.92, 86.7%, 90.3%, 88.1%). However, differences among the AUCs of ADC, CTRW parameters, and their combinations were not statistically significant(P=0.051–0.507).

**Table 4:** ROC analysis of the diagnostic performance for Dm, α, β, ADC, and their combinations in discriminating CP from PCa and low-graded PCa from high-grade PCa.

	α	β	Dm	Dm, α, β	Dm, α, β & ADC	ADC
CP(n=30) vs. PCa(n=31)						
Sensitivity (%)	83.3%	50.0%	83.3%	86.7%	90.0%	80.0%
Specificity (%)	77.4%	54.8%	87.1%	90.3%	87.1%	87.1%
Accuracy (%)	88.1%	50.0%	85.7%	88.1%	90.5%	85.7%
AUC (95% CI)	0.84(0.74,0.94)	0.50(0.35,0.65)	0.89(0.80,0.97)	0.92(0.86,0.99)	0.93(0.86,0.99)	0.86(0.76,0.96)
P-value	<0.001***	0.387	<0.001***	<0.001***	<0.001***	<0.001***
Low-graded PCa(n=15) vs. High-grade PCa(n=16)						
Sensitivity (%)	80.0%	53.3%	66.7%	73.3%	73.3%	66.7%
Specificity (%)	78.6%	50.0%	99.9%	93.7%	99.9%	92.9%
Accuracy (%)	78.9%	27.0%	82.8%	83.2%	86.2%	79.4%
AUC (95% CI)	0.86(0.71,0.99)	0.42(0.21,0.63)	0.85(0.71,0.98)	0.85(0.71,0.99)	0.87(0.72,0.99)	0.84(0.75,0.96)
P-value	<0.001***	0.453	<0.001***	<0.001***	<0.001***	<0.001***

***P-value<0.001.

### Diagnostic performance for differentiation between low-grade and high-grade PCa

The ROC analysis results are shown **Figure 4B** and **Table 4**. α exhibited the highest sensitivity, and AUC value (80.0% and 0.86), yet the specificity and accuracy were moderate (78.6% and 78.9%); Dm showcased the highest specificity, and accuracy (99.9% and 82.8%), followed by ADC (92.9% and 79.4%). β produced the lowest sensitivity, specificity, accuracy, and AUC (53.3%, 53.0%, 27.0% and 0.42). For the combination of parameters: (Dm, α, β) and (Dm, α, β & ADC) had the same sensitivity (73.3%); (Dm, α, β & ADC) had higher specificity, accuracy, and AUC (99.9%, 86.2% and 0.87), surpassing (Dm, α, β), which showed values of 93.7%, 83.2%, and 0.85, respectively. However, no statistically significant difference among AUCs of ADC, CTRW parameters, and their combinations in differentiating low-grade PCa from high-grade PCa lesions was found (P=0.059–0.526).

### The correlation between CTRW parameters with ADC and grade group

**Figure 5:**
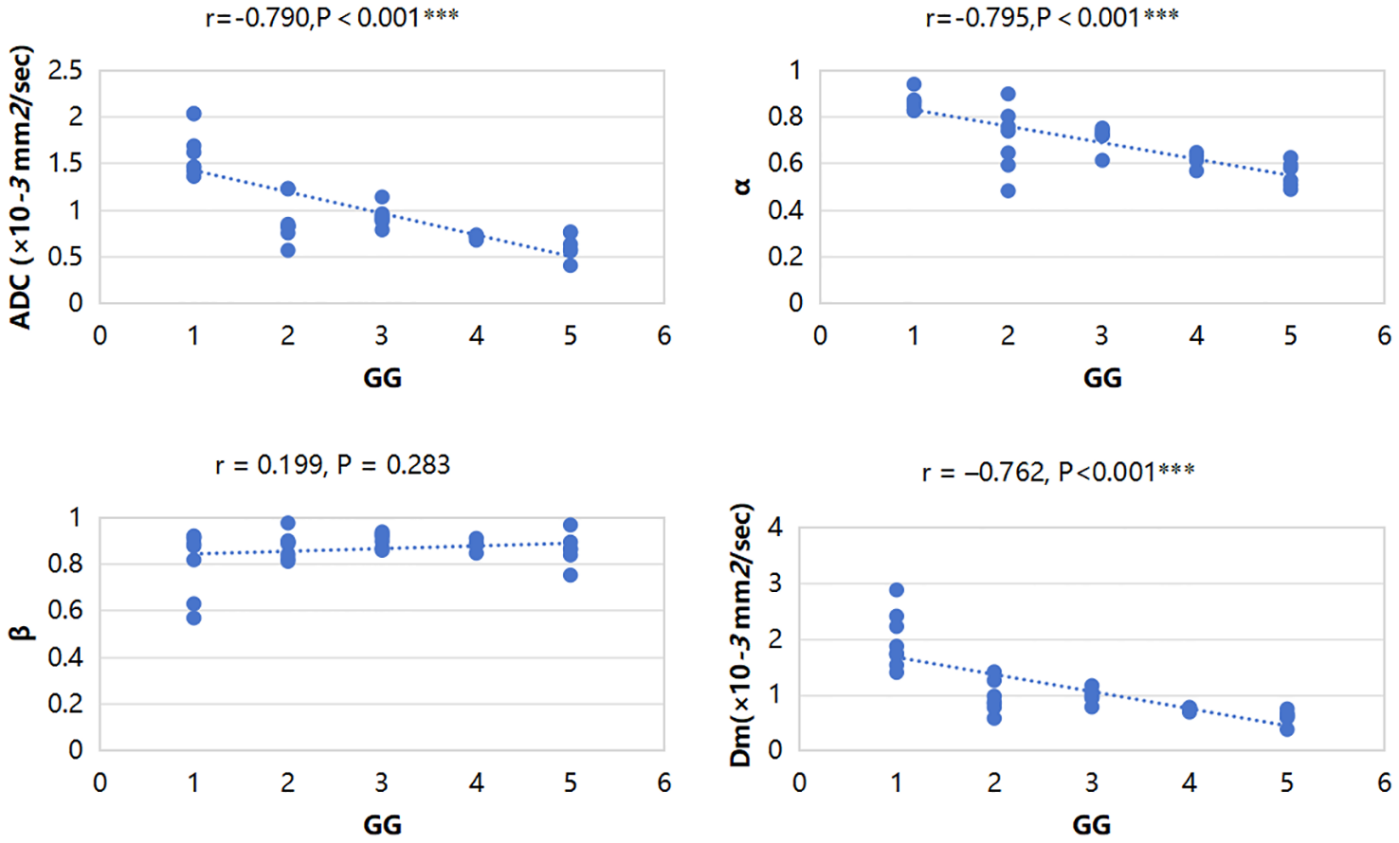
Scatterplots of the correlation between CTRW parameters, ADC and grade group, and ADC, α, and Dm were significantly negatively correlated with GG. ***P-value<0.001.

ADC, α, and Dm were significantly negatively correlated with GG (r = –0.790, P<0.001; r = –0.795, P<0.001 and r = –0.762, P<0.001, respectively). β showed no significant correlation with GG (r = 0.199, P = 0.283). The correlations between all parameters and GG are displayed in **Figure 5**.

## Discussion

The CTRW diffusion model has emerged as a promising DWI technique with wide-ranging clinical investigations such as breast tumors (19, 20), gliomas (15), hepatocellular carcinoma (21), Parkinson’s disease (22), obstructive sleep apnea (23), amyotrophic lateral sclerosis (24), etc. But to date, its application in identifying prostate cancer and assessing its grading remains unexplored. In this study, we demonstrated the feasibility of CTRW parameters in distinguishing PCa from CP and characterizing PCa grades, which indicated the CTRW model might hold promise for improving diagnostic accuracy and guiding treatment strategies for PCa patients.

The α value can reflect the temporal heterogeneity of the tissue by describing the probability that a water molecule will be retained or released as it diffuses through a tissue structure. Previous researches have demonstrated that lower α value corresponds to increased tissue heterogeneity (15, 17). In this study, α of PCa were significantly lower than that of CP, consistent with previous research theories. The histomorphological and molecular tumor characteristics of PCa exhibit considerable diversity, contributing to heightened structural complexity in the tissue microenvironment (11, 12). Therefore, water molecules may take drastically various periods of time to make a move (ie, temporal heterogeneity) within cancerous tissue (13, 14, 25). In contrast, CP is mainly proliferating inflammatory tissue and has low microenvironmental tissue complexity. For the ROC curve analysis, α had higher sensitivity and accuracy than ADC in differentiation between PCa and CP, which indicates that the parameter α of the CTRW model can provide information on tissue heterogeneity to help in the differential diagnosis of PCa and CP.

Dm describes the degree of dispersion of water molecules in the lesion, and this parameter is similar to the ADC parameter in traditional DWI (26), but ADC only describes the Gaussian diffusion behavior of water molecules (26). Dm parameters are sensitive to the structure of tumor cells, so the degree of malignancy can be determined by detecting the proliferation level of tumor cells (15, 16). In malignant tumors, due to the uncontrolled proliferation of cancer cells, the density of diseased cells is higher, the structure is more irregular, and the diffusion is more hindered and restricted, so the Dm and ADC values are significantly reduced (26). PCa tissue has higher cell density, small intercellular spaces, and complex microstructure, which restrict the diffusion of water molecules (11, 12). Our findings demonstrated significantly lower Dm values in PCa compared to CP, suggesting greater water molecule restriction in PCa tissue, which was consistent with previous research theories (7, 8, 27). Dm exhibited higher sensitivity and AUC values than ADC in diagnosis, indicating its potential diagnostic value in distinguishing between PCa and CP. Combining CTRW parameters (Dm, α, β) with ADC produced the highest sensitivity, diagnostic accuracy, and AUC value, while CTRW parameters (Dm, α, β) exhibited a high specificity, which indicated that the CTRW parameters can provide more comprehensive and complementary information on the spatial and temporal dimensions of the diffusive movements of water molecules in the heterogeneous tumor microenvironment that reflects neoplastic tissue changes (14, 28). ADC provided information on the Gaussian diffusion behavior of water molecules in prostate lesions (26), and the CTRW model provided relevant parameters of tissue heterogeneity (15). The combination of the two may hold the potential in more accurately identifying PCa and CP.

In diagnosing high-grade PCa via low-grade PCa, α exhibited the highest sensitivity, and Dm, α, β combined ADC provided the highest sensitivity and AUC. The Dm and ADC values were found to be significantly lower in high-grade PCa lesions compared to those in the low-grade PCa group. PCa of different grades, varying levels of proliferation, and diverse densities of cancer cells result in dissimilar microstructures within cancerous tissues, leading to differing degrees of abnormal water molecule diffusion (11, 12, 29). Additionally, high-grade PCa are associated with increased occurrences of necrosis, cysts, and bleeding, further contributing to tissue heterogeneity (30). α and Dm reflect temporal diffusion heterogeneity and anomalous diffusion coefficient, respectively. Consequently, the α, Dm, and ADC values for high-grade PCa are lower compared to those for low-grade PCa due to higher heterogeneity and a higher degree of restriction on water molecule dispersion. Our study revealed Dm had superior specificity and accuracy over ADC, indicating its potential in distinguishing between high and low-grade PCa. Moreover, Dm, α, β combined ADC had higher diagnostic efficacy, suggesting that the combination of water molecular dispersion information from traditional DWI and tissue heterogeneity information from the CTRW model can effectively improve the diagnostic efficiency of differential diagnosis of high- and low-grade PCa.

This study showed that the CTRW parameter α, Dm, and ADC were significantly negatively correlated with GG, suggesting that the CTRW parameter and ADC can indirectly reflect the grading of PCa. PCa exhibits significant heterogeneity at the molecular, cellular, and tissue levels, and the diffusion of water molecules is significantly limited. As the grade of PCa increases, tissue heterogeneity and the degree of diffusion limit of water molecules also increase (5, 27, 31). Levels of hemorrhage, necrosis, and abnormal diffusion of water molecules are more pronounced in high-grade pCa tissues compared to low-grade pCa tissues (32, 33). Thus, lower values of α, Dm, and ADC tended to correlate with higher-grade PCa.

β reflects the heterogeneity of the diffusion “jump” lengths in each movement (14), many previous studies have shown that β has good efficacy in distinguishing benign and malignant breast tumors, high- and low-grade glioma in adults, and high- and low-grade brain tumors in children (15, 16, 28). This study showed that the lack of significant difference in β may be related to the small sample size, and may also be due to the tissue characteristics of the prostate itself, which needs further investigation in larger independent cohorts.

Although this study had some insights, it still had some limitations. First of all, this study chose the largest tumor section when mapping the ROI target area, which may ignore the information of other sections. Second, this study is a single-center study with a small sample size, which may lead to uneven data distribution and failure to obtain an effective verification cohort. Third, though the DWI scan time was acceptable (7 minutes and 10 seconds), but needed to be further optimized to select fewer, more appropriate B-values. Finally, this study lacked an analysis of zonal distribution in prostate lesions due to the small sample size of PCa and CP (31 and 30 cases, respectively). Further studies in a larger cohort are required to focus on the CTRW model performance in prostate lesions with different zonal distribution.

In conclusion, Both the CTRW model and ADC could effectively differentiate PCa from CP and high-grade PCa from low-grade PCa, and CTRW parameters and ADC showed comparable diagnostic performance. Although no significant difference was found in diagnostic efficiency among ADC, CTRW parameters and the combination model, the highest diagnostic performance was obtained by the integration of CTRW model with conventional ADC, which implies its potential value in providing comprehensive information and facilitating accurate characterization of PCa.

## Data availability statement

The raw data supporting the conclusions of this article will be made available by the authors, without undue reservation.

## Ethics statement

The studies involving humans were approved by The First Affiliated Hospital of Shandong First Medical University & Shandong Provincial Qianfoshan Hospital, Ethics Committee. The studies were conducted in accordance with the local legislation and institutional requirements. The participants provided their written informed consent to participate in this study.

## Author contributions

YS: Writing – review & editing, Writing – original draft, Visualization, Validation, Supervision, Software, Project administration, Methodology, Investigation, Formal analysis, Data curation, Conceptualization. HC: Writing – original draft, Data curation. KX: Writing – review & editing. JC: Writing – original draft, Investigation. TJ: Writing – original draft, Software. DC: Writing – original draft, Software. HW: Writing – original draft, Methodology. GZ: Writing – original draft, Methodology. YY: Writing – original draft, Methodology. QZ: Software, Investigation, Formal analysis, Data curation, Writing – review & editing, Methodology.
